# Propagation of THz radiation in air over a broad range of atmospheric temperature and humidity conditions

**DOI:** 10.1038/s41598-023-47586-8

**Published:** 2023-11-27

**Authors:** Fatima Taleb, Mariana Alfaro-Gomez, Mohanad Dawood Al-Dabbagh, Jan Ornik, Juan Viana, Alexander Jäckel, Cornelius Mach, Jan Helminiak, Thomas Kleine-Ostman, Thomas Kürner, Martin Koch, Daniel M. Mittleman, Enrique Castro-Camus

**Affiliations:** 1 Department of Physics and Material Sciences Center, Philipps-Universität Marburg, Renthof 5, 35032 Marburg, Germany; 2 Universidad Autonoma de Aguascalientes, Av. Universidad 940, Cd. Universitaria, 20100 Aguascalientes, Mexico; 3 Physikalisch-Technische Bundesanstalt (PTB), 38116 Braunschweig, Germany; 4 Institut für Nachrichtentechnik, Technische Universität Braunschweig, 38106 Braunschweig, Germany; 5 School of Engineering, Brown University, 184 Hope Street, Providence, RI 02912 USA

**Keywords:** Electrical and electronic engineering, Terahertz optics

## Abstract

As the need for higher data rates for communication increases, the terahertz (THz) band has drawn considerable attention. This spectral region promises a much wider bandwidth and the transmission of large amounts of data at high speeds. However, there are still challenges that need to be addressed before the THz telecommunications technology hits the consumer market. One of the recurring concerns is that THz radiation is greatly absorbed by atmospheric water-vapor. Although many studies have presented the attenuation of THz signals under different atmospheric conditions, these results analyze specific temperature or humidity values, leaving the need for a more comprehensive analysis over a wider range of climate conditions. In this work, we present the first study of the attenuation of THz radiation over a broad range of temperatures and humidity values. It is worth noticing that all of our measurements have been undertaken at atmospheric pressure unlike many previous studies where the pressure was not kept constant for various temperatures. Furthermore, we extend our analysis beyond the impact of absolute humidity on the bit error rate in THz communications. We also discuss the refractivity of the atmosphere, examining its variations across different temperatures and humidity levels. THz propagation is studied using two different measurement systems, a long-path THz time-domain spectrometer as well as a quasi-optic setup with vector network analyze. We also compare the results with the ITU-R P.676-13 propagation model. We conclude that the attenuation at the absorption peaks increases linearly with water content and has no dependence on the temperature, while the refractive index, away from absorption lines, namely at 300 GHz shows a sub-linear increase with humidity.

## Introduction

The way we communicate has been radically changed by the wireless transmission of information. The need for higher data rates continuously pushes the development of new generations of communication technologies with improved performance. In this context, it has been predicted for many years that terahertz (THz) wireless communication above 100 GHz, would fulfill the needs of data transmission in the future^1^. The THz band presents intrinsic advantages over other spectral bands such as increased bandwidth and improved security^2–7^. However, there are still challenges that have to be addressed before this technology can be completely deployed for the consumer market^8–10^. In particular, the potential effects of the atmospheric conditions on the transmission channels are among the concerns for THz data transmission due to the high absorption by water vapor at certain frequencies^11,12^. Indeed, many studies on the characterization of the attenuation of the THz signal under different atmospheric conditions have been reported^10,12–20^. However, these results are normally presented as a function of humidity for a fixed temperature, and generally over a fairly narrow range of values. Transmission of THz signals carrying data would generally not occur over such restricted conditions. Any realistic approach should consider that both variables may change independently and in wider ranges in real scenarios.

Whereas such considerations are less relevant for short-range links^9^ such as indoor settings^21^, long-range outdoor links are becoming increasingly relevant, with the growing interest in backhaul as one of the first key commercial implementations of links above 100 GHz^22,23^. Furthermore, the properties of the atmosphere offer unique opportunities in the THz band that are not available to communication systems operating at lower frequencies. For instance, one can exploit the attenuating properties of the atmosphere for enhanced security against eavesdropping^4^. Also, the spectral filtering effects of absorption peaks of water molecules suggests a unique encoding strategy for multiple access via hierarchical bandwidth modulation^24^. Thus, it is relevant to have a highly accurate characterization of the propagation of THz radiation for a continuous range of temperature and humidity as wide as possible. To address these issues, we implement control of environmental conditions using a climate chamber with an internal propagation path length of 6 m and 10.9 m for THz-TDS and VNA respectively, in order to analyze THz attenuation over a wide range of temperature and humidity. Our measurements have been undertaken entirely in atmospheric pressure conditions, unlike many previous absorption studies where the pressure was not kept constant as the temperature was varied^25–28^.

We employ two measurement techniques to improve the robustness of our results. First, we characterize the THz pulse propagation using a long-path THz time-domain spectrometer (THz-TDS) which has been shown to be a viable method for studying long-path propagation over a broad bandwidth^18,19,29^. Second, we use a vector network analyzer (VNA) to measure the CW frequency-dependent attenuation of air^30^. From the analysis of the THz attenuation, we calculate the impact of the absolute humidity on the bit error rate (BER) in a THz communication link. In addition, we present the variation of refractivity of the atmosphere for different temperature and humidity levels. Our results allow better understanding of the THz propagation channels for a wide range of possible climate scenarios, which in turn is important for the design of THz communication systems.

## Experimental methods

### A climate chamber

**Figure 1:**
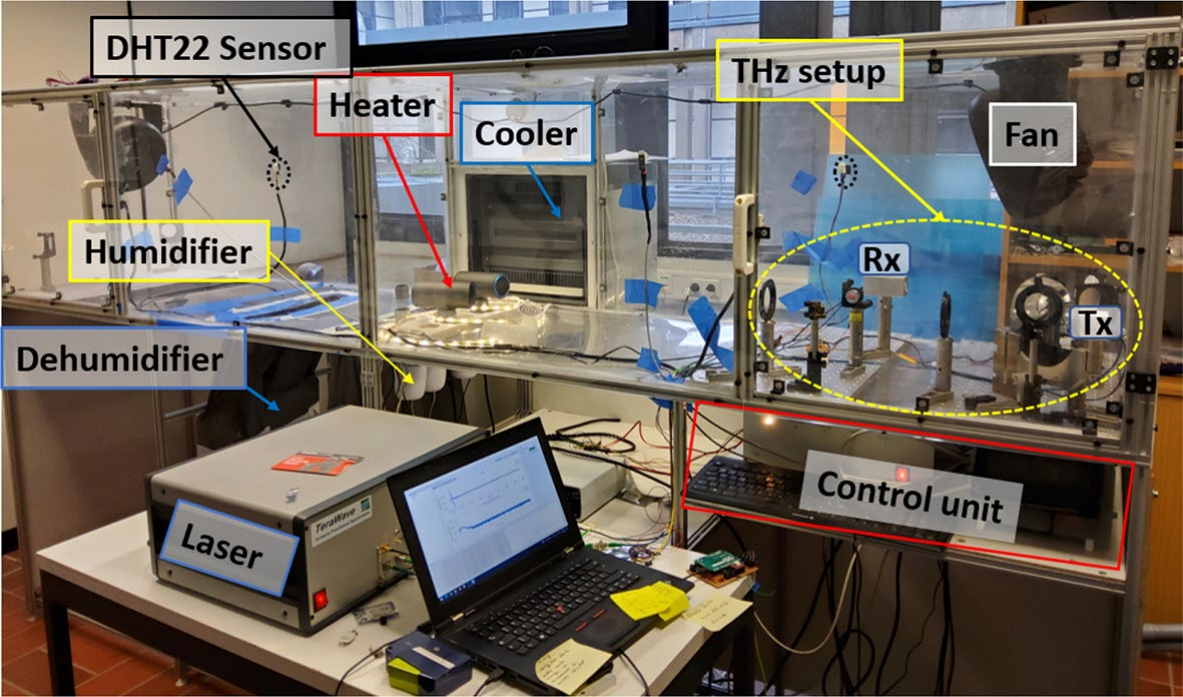
Climate chamber used to control humidity and temperature. Inside the chamber two wire-coil heaters and a cooler change the temperature. Four humidifiers and a dehumidifier vary the absolute humidity. The THz radiation from the TDS and the VNA systems propagates within the chamber at a wide range of climate conditions. The picture is shown with the TDS system. Photograph by Fatima Taleb.

We constructed a climate chamber that allows us to maintain an atmosphere with controlled temperature and humidity conditions^4,13,14,31,32^. Our climate chamber (Figs. 1 and 4) has dimensions of 3 m $\times$ 0.6 m $\times$ 0.6 m. The walls of the chamber are two layers of Plexiglas with an insulating air gap of 2 cm between them. All edges are sealed with silicone. Inside the chamber, four humidifiers and one dehumidifier are used to control the absolute humidity while two wire-coil heaters and a cooler are used to control the temperature. Each of these devices is controlled by a Raspberry PI 4. Two DHT22 sensors, located on each side of the chamber, measure the relative humidity (RH) and temperature with an accuracy of $\pm 2$% and $\pm 0.5$  °C, respectively. The relative humidity and temperature values were acquired every two seconds. The Raspberry controller turns on and off each device after reading the sensors to reach or maintain the desired chamber conditions. We use two fans on each side of the chamber to circulate air at low speed during the measurements, in order to assure a uniform distribution of temperature and water-vapor over the entire chamber. Although this may create a small amount of atmospheric turbulence during the measurements, it has previously been demonstrated that THz beams are relatively insensitive to such mild levels of turbulence^32^, so we do not expect that this procedure has a significant impact on the measurement results, since the signals measured under constant conditions in the chamber showed a power fluctuation of 1 part in $10^4$ for THz-TDS and 0.05 dB for the VNA S-parameters.

**Figure 2:**
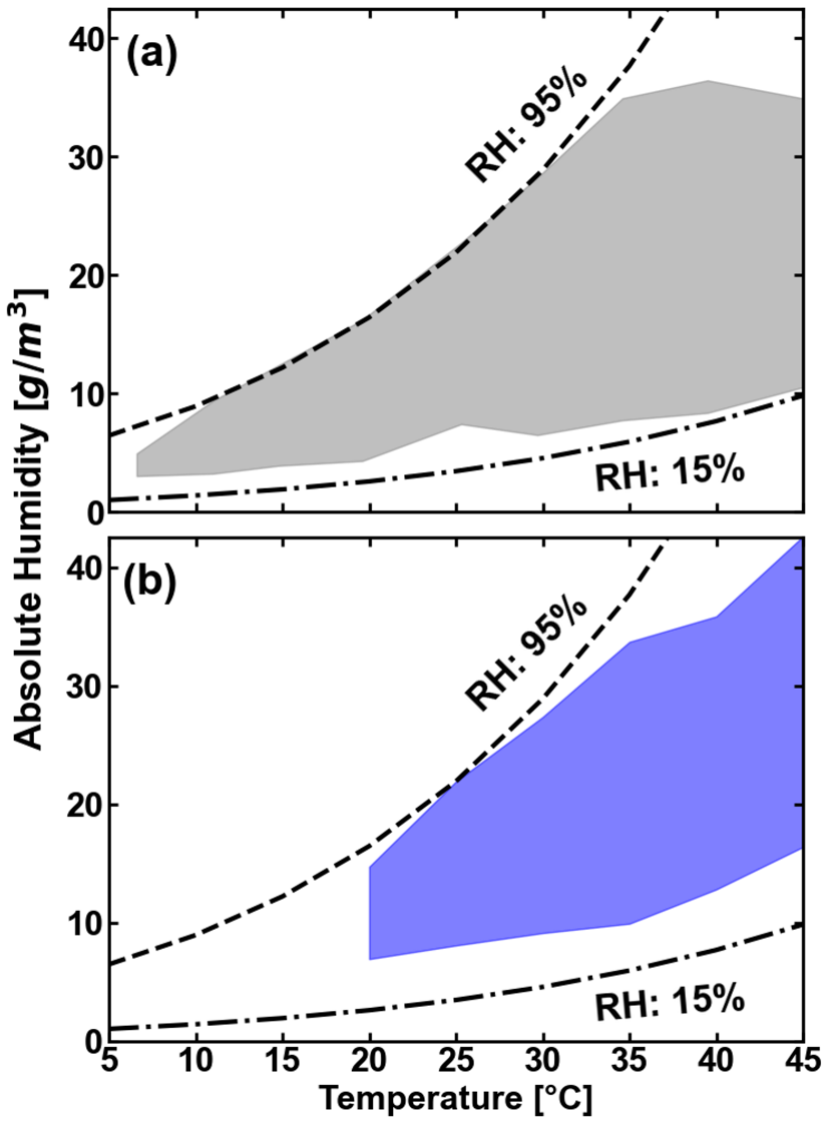
Temperature and absolute humidity regions covered in the study. (**a**) In the THz-TDS experiment, temperature change at ±5 °C steps over an interval of 6 °C to 45 °C. Absolute humidity values range from 2.7 to 36.9 g/m$^3$. Each temperature value is fixed while the relative humidity change with step of $\pm 5$%. For each TDS measurement, the climate chamber is stabilized at the corresponding humidity-temperature pair. (**b**) For the VNA experiment, the covered temperature interval ranges from 20 to 45 °C with ± 5 °C steps. The absolute humidity values in this setup can be varied from 7.5 to 40.5 g/m$^3$.

**Figure 3:**
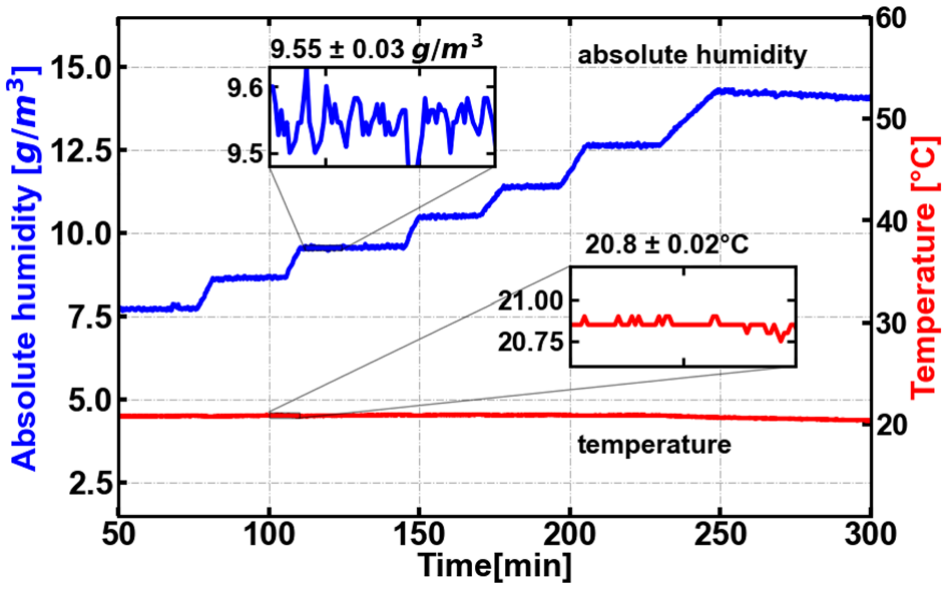
The absolute humidity changes from 7.5 to 13.25 g/m$^3$ while the temperature is fixed at 20 °C. The stability of the humidity and temperature is depicted in the insets. The small oscillations are a result of feedback of the temperature/humidity control system.

Temperature and relative humidity are changed for the measurements in $\pm 5$  °C and $\pm 5$% RH steps over an interval of 6 °C to 45 °C and 20% to 95% of relative humidity, corresponding to 2.7 g/m$^3$ to 36.9 g/m$^3$ of absolute humidity over the total interval of temperatures that we studied. These values are calculated using the ideal gas equation and Tetens formula for saturation vapor pressure^33^. Fig. 2a shows the range of atmospheric conditions covered in the THz-TDS experiment, while Fig. 2b depicts the explored range in our VNA measurements. During the measurements, the temperature of the chamber is fixed while the relative humidity value is adjusted and then allowed to stabilize. At each stabilized humidity value, a THz measurement is made. The THz signals are registered ten minutes after the corresponding value of absolute humidity and temperature is reached, allowing the chamber ambient conditions to fully equilibrate. Figure 3 shows the absolute humidity changes for a fixed value of 20 °C, as an illustration of the degree of control of both variables that we can achieve in the climate chamber.

We emphasize that our chamber is not hermetically sealed from the external atmosphere. As a result, when we vary the temperature of the air inside the chamber, the pressure is able to equalize to the external atmospheric pressure. As a result, unlike most previous published examples of studies of atmospheric transmission in the THz band^16,25–28^, our measurements at different temperatures can be compared to each other fairly because the pressure remains constant, while the temperature is varied. In this regard, our data are unique in comparison with most earlier studies. The atmospheric pressures were $974\pm 12$ mbar and $1027\pm 4$ mbar at the time of the THz-TDS and VNA measurements respectively.

### Long-path THz time-domain spectrometer (THz-TDS)

**Figure 4:**
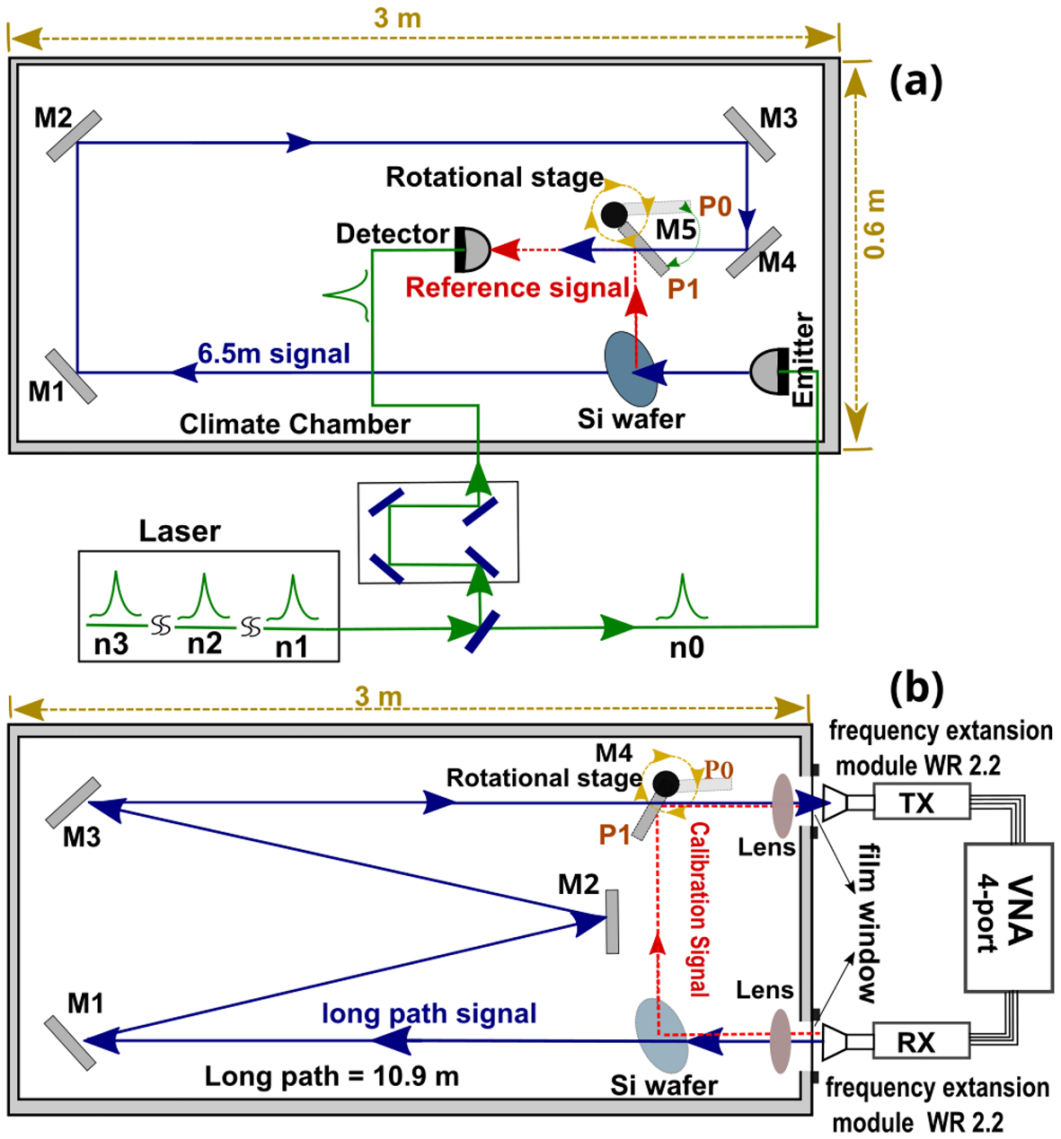
(**a**) Fiber-coupled THz-TDS system and (**b**) VNA system for atmospheric measurements within the controlled humidity and temperature chamber. Red (reference) and blue (sample) paths represent free air propagation of the THz radiation within the chamber. Green paths indicate laser pulse propagation in optical fiber. Schematics drawn by Fatima Taleb.

**Figure 5:**
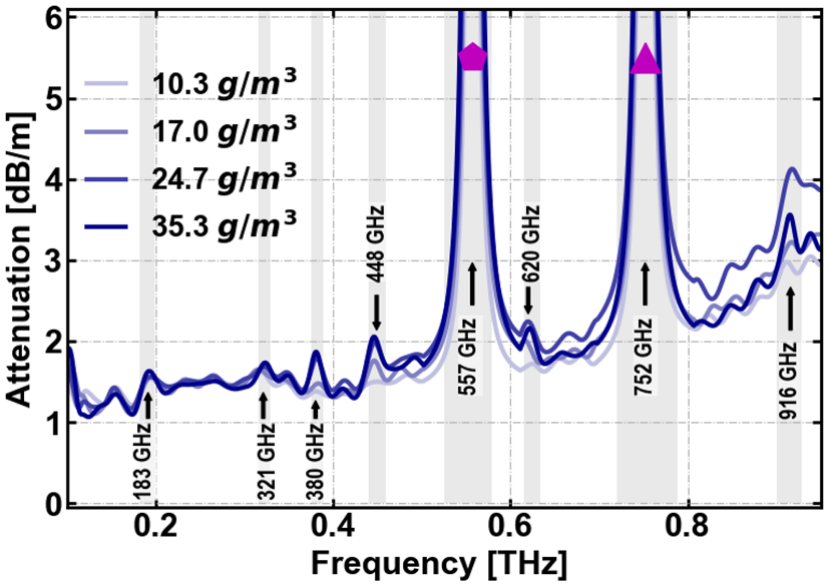
Absorption spectra at 45 °C corresponding to 10.3 g/m$^3$, 17 g/m$^3$, 24.7 g/m$^3$, 35.3 g/m$^3$ of absolute humidity. Strong resonant water-vapor lines are marked in pale-gray. The polygon and triangle symbols mark the analyzed frequencies shown at 557 GHz and 752 GHz (Fig. 6a and b).

The THz-TDS system uses an infrared (1550 nm) 80 fs pulsed laser with a 100 MHz repetition rate. The laser pulse is split into the detection and emission arms. LT-InGaAs photo-conductive antennas generate and detect the THz signal. A high density polyethylene (HDPE) lens collimates the emitted THz radiation and the signal propagates 6.54 m before detection by using mirrors M1 to M4, as shown in Fig. 4a. Through this path, the beam-waist diameter expands from (11.5 ± 0.9) mm to (32 ± 1.5) mm. The total loss of all optical components is calculated to be 13$\%$ or 0.6 dB. A silicon wafer in the THz path is used as a THz beam splitter at 45°, generating a reflected THz reference signal that propagates over a 0.54 m distance to the detector. The Si-wafer is also used for the alignment of the system. The 6 m propagation distance corresponds to 2 round-trips of the laser pulse in the laser cavity, therefore the detected THz radiation is delayed by 2 repetition rate cycles with respect to the excitation pulse. THz pulses are measured over a 200 ps window which corresponds to a 5 GHz frequency resolution. This spectral resolution is not sufficient to resolve the line shapes of pressure-broadened absorption lines, so our analysis does not include results related to the line widths. The spectra presented in Figs. 5 and 10c were obtained by additionally applying a window of 60 ps around the main pulse in order to remove Fabry-Pérot oscillations caused by internal reflection in the optical or photoconductive components. Further, since the peak values of the two stronger absorption lines at 557 GHz and 752 GHz were in many cases larger than the dynamic range of our measurement system, we have employed an interpolation fitting procedure to characterize the central parts of these lines.

Given that the THz long/short path is directed by mirrors placed over aluminum boards and fixed on aluminum posts, the thermal dilation must be considered in the analysis. The thermal expansion coefficient ($\Delta L/L$)/ °C of aluminum is $23\times 10^{-6}/$ °C ^34^. Then, for a temperature difference of $\Delta T$ = 5 °C, a length difference of 0.7521 mm and 0.0621 mm affects the optical path of the long and short pulses, respectively. This corresponds to a time difference of 2.51 ps for the long path pulse and 0.39 ps for the short path. The start time of each analyzed THz pulse is therefore shifted back by this amount of time, in order to take into account the corresponding thermal shift.

In order to measure the attenuation of the THz pulse at different atmosphere conditions, each value of the temperature and absolute humidity in the chamber is set and stabilized (Section “A climate chamber”). The reference signal, which is reflected from the silicon wafer and travels a shorter path, is measured with mirror M5 rotated to position P1 (Fig. 4a), reflecting the reference signal to the detector. Then, mirror M5 is rotated to position P0, so that the detector measures THz pulses which have traversed the longer path. At each humidity-temperature pair, we average 50 pulses, and the total measurement lasts approximately 30 s. Reference and signal are measured 5 times and are averaged for each humidity-temperature pair. The detected signal is zero-padded to a total scan length of 1000 ps prior to Fourier analysis.

### Vector network analyzer

We also implement a second set of experiments using the same climate chamber with a different quasi-optical configuration. A calibrated Rohde & Schwarz ZVA 50 vector network analyzer measures the reflection and transmission coefficients (S-parameters) of the quasi-optic setup under different climate conditions. We used a 4-port VNA with WR-2.2 VNAX frequency extension modules and two standard gain horn antennas of 20 dBi. Figure 4b shows the VNA experimental setup. In this case, unlike the TDS measurements, the receiver (RX) and transmitter (TX) are located outside the climate chamber. Two windows of polyethylene film allow the propagation of the signals inside. The Si-wafer acts as a mirror to deviate the signal through the calibration path (red in the figure). The rotational stage sets the position of mirror M4 to measure calibration (P1) or signal (P0) data. The long path signal travels 10.9 m reflecting on mirrors M1 to M3 before reaching the receiver. For these measurements, the Si-wafer is removed. The beam-waist diameter expands from (24 ± 1.1) mm to (65 ± 1) mm along this path. The total loss of all optical components is calculated to be 28$\%$ or 1.3 dB. Temperature and absolute humidity are varied within the climate chamber as previously described (Section “A climate chamber”). These experiments are performed with the climate chamber located inside an anechoic chamber with a constant temperature of 22 °C and RH of 50%. The temperature and absolute humidity map covered with this analysis is depicted in Fig. 2b. The range cover a part of the conditions studied with TDS allowing us to compare the results obtained with each technique. The measurements that we will discuss further are centered on absorption lines at frequencies 380.2 GHz and 448.0 GHz with a resolution of 100 MHz over a frequency range of 2 GHz around the peak center.

By sampling incident, transmitted, and reflected waves, the S-parameters are determined for each frequency^35^. We use the UOSM (Unknown Through, Open, Short, Match) calibration technique, to determine and correct for systematic measurement errors such as impedance mismatch, system drift, or instrument noise at the waveguide flanges. This procedure involves measuring a set of calibration standards to determine error coefficients, which can then be used to transform the raw S-parameters of a measurement into corrected S-parameters^36^.

## Data processing

### THz time-domain data

When analyzing the THz pulse transmission through a free-space path, the spectral attenuation and absorption are dominated by the resonant absorption lines of water vapor. The climate conditions, in particular the value of the absolute humidity and temperature, should then determine the transmission loss through the atmosphere from a transmitter to a receiver, in a given frequency range. For such a line-of-sight model, the complex transfer function of the system is $\tilde{H}=\tilde{E_L}/\tilde{E_S}$, where $\tilde{E_L}$ and $\tilde{E_S}$ are the Fourier Transforms of the Long and Short paths followed by the corresponding THz electric field, $E_L$ and $E_S$. Theoretically, for our experimental setup, $\tilde{E_S}$ is calculated with

1 $$\begin{aligned} \tilde{E_S}=E_0r_{21}t_{12}t_{21}e^{-2iK\tilde{n}_{Si}d_{Si}}e^{-ikn_{air}d_{R}}, \end{aligned}$$

where $E_0$ is the incident pulse, $r_{21}$, $t_{12}$ and $t_{21}$ are the corresponding Fresnel coefficients for reflection and transmission between Si-air interfaces at the corresponding incidence angle, and *K*, *k* are the wave-vectors in silicon and air, respectively. $d_{Si}$ is the Si wafer thickness, $n_{Si}$ is the refractive index of Si and $d_{R}$ refers to the traveling distance through air. The refraction of the pulse for the 45° incidence angle is considered for the calculation of $d_{Si}$. The reference pulse is reflected at the posterior face of the silicon wafer and travels a distance of 0.54 m before arriving at the detector.

On the other hand, $\tilde{E_L}$ is described by

2 $$\begin{aligned} \tilde{E_L}=E_0t_{12}t_{21}e^{-iK\tilde{n}_{Si}d_{Si}}e^{-ikn_{air}d_{n+2}} \end{aligned}$$

where $d_{n+2}$ equals the 6.54 m long path within the spectrometer after passing through the Si-wafer.

The propagation of an electromagnetic wave through the atmosphere depends on the index of refraction of water vapor, or refractivity $(n_{air}-1)$ and on the absorption coefficient $\alpha (\omega )$. The effect of different temperature and humidity conditions on THz propagation can be analyzed from both parameters. The refractivity can be calculated from the phase shift $\phi (\tilde{H)}$ of the transfer function, obtaining

3 $$\begin{aligned} n_{air}(\omega )-1=\frac{1}{\Delta d}\bigg [\frac{c\phi (\tilde{H})}{\omega }\bigg ]. \end{aligned}$$

here $\omega$ is the frequency and $\Delta d$ is the distance difference between short and long paths. The refractivity of humid air (Eq. 3) has two components: a frequency-independent part and a strongly frequency-dependent part at resonant water lines. The refractivity of the air at different atmosphere conditions has been previously described with the Essen and Froom formulation^37^ and from van-Vleck Weisskopf theory^38,39^.

The absorption coefficient of the atmosphere can be calculated from the magnitude $|\tilde{H}|$ of the transfer function,

4 $$\begin{aligned} \alpha (\omega )=\bigg (\frac{-2}{\Delta d}\bigg )\bigg [ln\big (r_{21}|\tilde{H}|\big )-\frac{1}{2}\alpha _{Si}d_{Si}\bigg ] \end{aligned}$$

where $\alpha _{Si}$ is the absorption coefficient of Si. The attenuation in dB/m can be calculated as

5 $$\begin{aligned} attenuation_{TDS} = \frac{20log_{10}(e^{\alpha (\omega )\Delta d/2})}{\Delta d_{TDS}}. \end{aligned}$$

This quasi-optic analysis assumes that the diameter of the THz beam is small when compared to the size of every relevant optical component (mirrors, lenses, beam splitter, etc.) so that the losses due to overfilling of any component can be neglected. We also neglect the small frequency-independent loss of 0.6 dB due the optical components, mentioned in section “Long-path THz time-domain spectrometer (THz-TDS)”.

### VNA analysis of S-parameters

The frequency-dependent complex-valued S-parameters are commonly used in radio-frequency and microwave measurements^35,36^. They are related to the transmission and reflection of a high-frequency electromagnetic signal interacting with, in our case the atmosphere. From the measured S-parameters, the attenuation can be obtaind as

6 $$\begin{aligned} attenuation_{VNA} = \frac{-20log_{10}\Big (\Big |S_{21}\Big |\Big )}{d_{VNA}}. \end{aligned}$$

here $d_{VNA} =$ 10.9 m and $S_{21}$ is the transmission coefficient. From Eqs. (5) and (6), it is possible to compare the attenuation of the THz radiation obtained with each experimental technique. The results are compared in the next section.

## Results and discussion

### Attenuation of THz radiation

**Figure 6:**
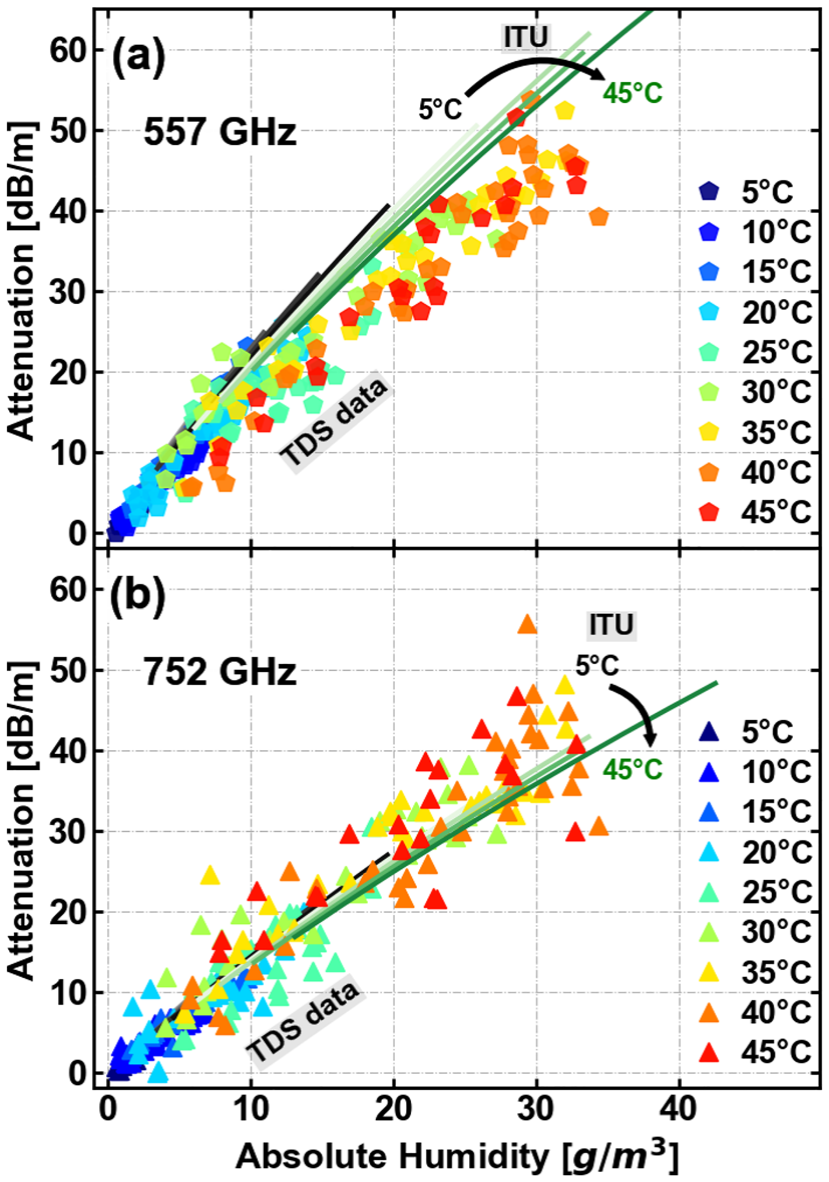
Attenuation of the absorption peaks at (**a**) 557 GHz and (**b**) 752 GHz for a temperature range from 5 to 45 °C with a 5° step, corresponding to a change in absolute humidity of 0 g/m$^3$ to 35 g/m$^3$. The attenuation at the peak increases linearly at a ratio of (1.438 ± 0.379) [dB/m]/[g/m$^3$] in (**a**) and (1.307 ± 0.441) [dB/m]/[g/m$^3$] in (**b**) for the corresponding water rotational lines. The attenuation values calculated with ITU-R P.676-13 model are represented by black (lower temperature) to green (higher temperature) lines for each frequency. The attenuation at the absorption lines presents a strong dependence on humidity.

Based on our measurements, we obtain the attenuation dependence of the THz radiation on the temperature and humidity. It is well known that the atmospheric absorption contains a number of very well localized absorption lines, almost all associated with rotational modes of the water molecule, superimposed on a featureless continuum also associated with the presence of water, but its precise physical origin is less well understood^40^. Our analysis primarily focuses on the water vapor lines, rather than the continuum absorption, as the change in continuum absorption is a minor perturbation within the transparent windows. For the propagation distance studied here, it falls within the systemic uncertainty of our measurement systems. The attenuation in dB/m obtained from the TDS measurements are presented in Fig. 5 for increasing absolute humidity at the same temperature (45 °C). The observed water-vapor lines in the absorption spectrum are marked in pale gray. We analyze the dependence of attenuation on temperature and humidity at the two higher water vapor lines at 557 GHz and 752 GHz. These frequencies are marked in Fig. 5 with a pentagon and a triangle, respectively. Figure 6 shows the attenuation for a variety of values of the temperature and absolute humidity. With increasing absolute humidity, the attenuation at the peak increases linearly. The rate of increase equals (1.438 ± 0.379) [dB/m]/[g/m$^3$] for the 557 GHz line (Fig. 6a) and (1.307 ± 0.441) [dB/m]/[g/m$^3$] for the 752 GHz line (Fig. 6b). These values compare reasonably well with those obtained from the ITU-R P.676-13 model^41^, which are (1.719 ± 0.252) [dB/m]/[g/m$^3$] for the 557 GHz line and (1.165 ± 0.154) [dB/m]/[g/m$^3$] for the 752 GHz line. The ITU-R P.676-13 model, which is an empirical fit, is a standard reference for estimation of the propagation of signals for the telecommunications community. This model estimates the attenuation for the band up to 1 THz, due to dry air and water vapour,considering the superposition of the individual spectral lines from oxygen and water vapour, and additional factors for the non-resonant Debye spectrum of oxygen, pressure-induced nitrogen attenuation and a continuum function to account for the excess water vapour-absorption observed experimentally are included.^41^

**Figure 7:**
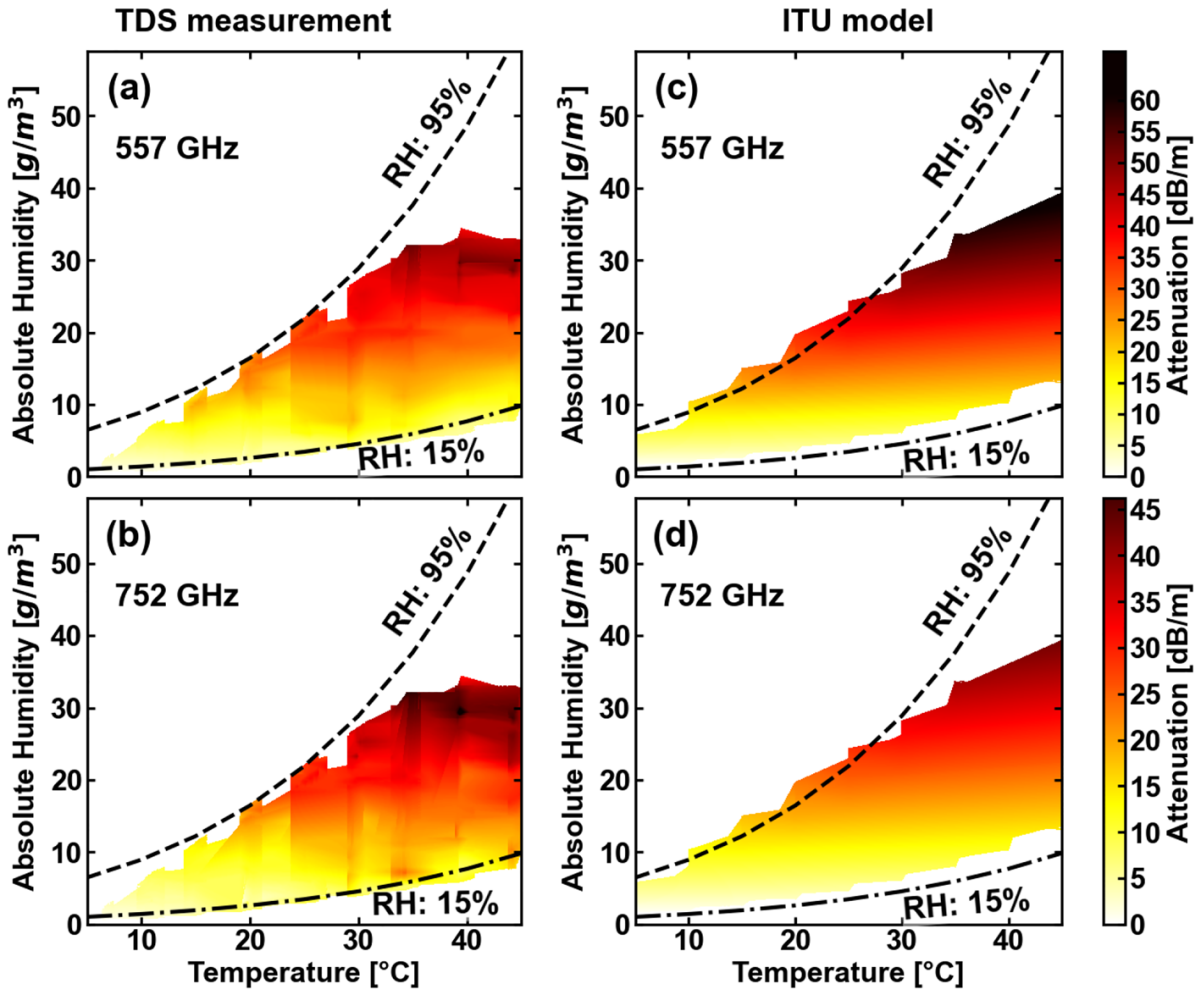
Temperature-humidity attenuation maps for the absorption lines at 557 GHz and 752 GHz as measured with (**a**) and (**b**) THz-TDS experiments, and (**c**) and (**d**) calculated with the ITU-R P.676-13 model. The experimentally obtained attenuation agrees with the theoretical attenuation calculated with the ITU-R P.676-13 model. The dashed lines represent the highest (95%) and lowest (15%) measured values of the relative humidity (RH).

Given the temperature and humidity intervals of our TDS experiments, we are able to present attenuation maps for the absorption peaks at 557 GHz (Fig. 7a) and 752 GHz (Fig. 7b). These maps can be compared with the corresponding attenuation obtained from the ITU-R P.676-13 propagation model (Fig. 7c and d)^41^. The lowest (15%) and highest (95%) values of the relative humidity reached in the experiment are represented with dashed lines. The trend of attenuation with temperature and humidity is similar for both frequencies. Specifically, the results show that the attenuation values remain relatively constant over a wide temperature range when the absolute humidity does not vary. These findings are consistent with the attenuation calculated from the ITU-R P.676-13 model, as seen in Figs. 7c and d.

**Figure 8:**
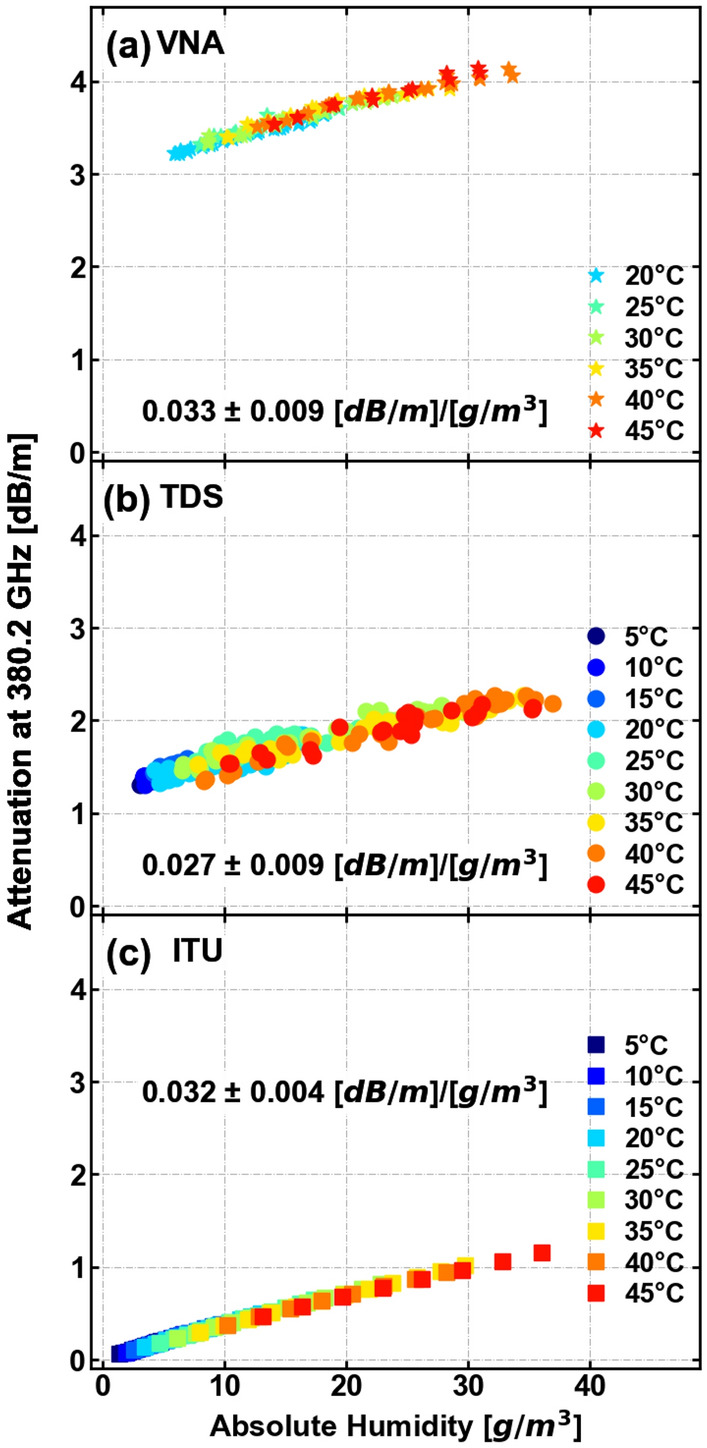
Attenuation for the absorption peak at 380 GHz measured by (**a**) VNA, (**b**) THz-TDS and (**c**) ITU-R P.676-13 model. The temperature interval is from 20 to 45 °C for VNA, and from 5 to 45 °C for THz-TDS and ITU-R P.676-13 model. The attenuation at the peak increases linearly at a ratio of (0.033 ± 0.009) [dB/m]/[g/m$^3$] in (**a**), (0.027 ± 0.009) [dB/m]/[g/m$^3$] in (**b**) and (0.032 ± 0.004) [dB/m]/[g/m$^3$] in (**c**). The dependence of attenuation with respect to absolute humidity is confirmed for both measuring techniques and ITU-R P.676-13 model.

**Figure 9:**
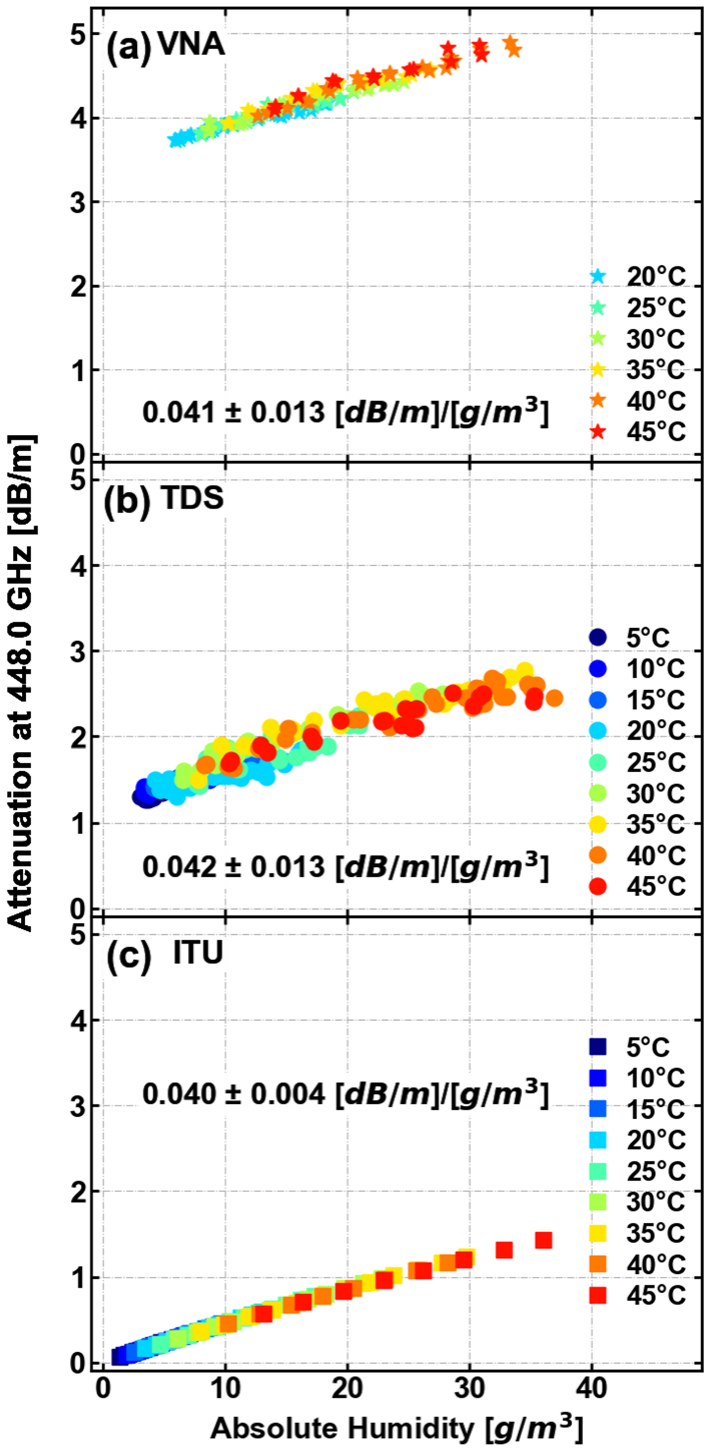
Attenuation for the absorption peak at 448 GHz measured by (**a**) VNA, (**b**) THz-TDS and (**c**) ITU-R P.676-13 model. The temperature interval is from 20 to 45 °C for VNA, and from 5 to 45 °C for THz-TDS and ITU-R P.676-13 model. The attenuation at the peak increases linearly at a ratio of (0.041 ± 0.013) [dB/m]/[g/m$^3$] in (**a**), (0.042 ± 0.013) [dB/m]/[g/m$^3$] in (**b**) and (0.040 ± 0.004) [dB/m]/[g/m$^3$] in (**c**). The dependence of attenuation with respect to absolute humidity is confirmed for both measuring techniques and ITU-R P.676-13 model.

Similar trends are observed for the less intense absorption peaks also present in the frequency band we studied. It is of interest to analyze different peaks of water-vapor absorption as it can be used to limit the broadcast range of a signal at those carrier frequencies, thereby serving as a countermeasure against potential eavesdroppers^4^. In particular, we now focus on the 380 GHz and 448 GHz peaks. For these absorption peaks, we can compare the results obtained with TDS and VNA experimental techniques. Figures 8 and 9 show the attenuation from (a) the VNA, (b) the THz-TDS and (c) the ITU-R P.676-13 model. We see that the attenuation is independent of the temperature and increases when absolute humidity is increased. The rate of change in attenuation with respect to humidity for the 380 GHz and 448 GHz lines is presented in each corresponding sub-figure.

The attenuation rates exhibit similar values and are similar to the results from the ITU-R P.676-13 model for both bands. The offset in attenuation values among the three methods as a function of absolute humidity can be attributed to a misalignment during the experiment and other systematic errors.

### Bit error rate

**Figure 10:**
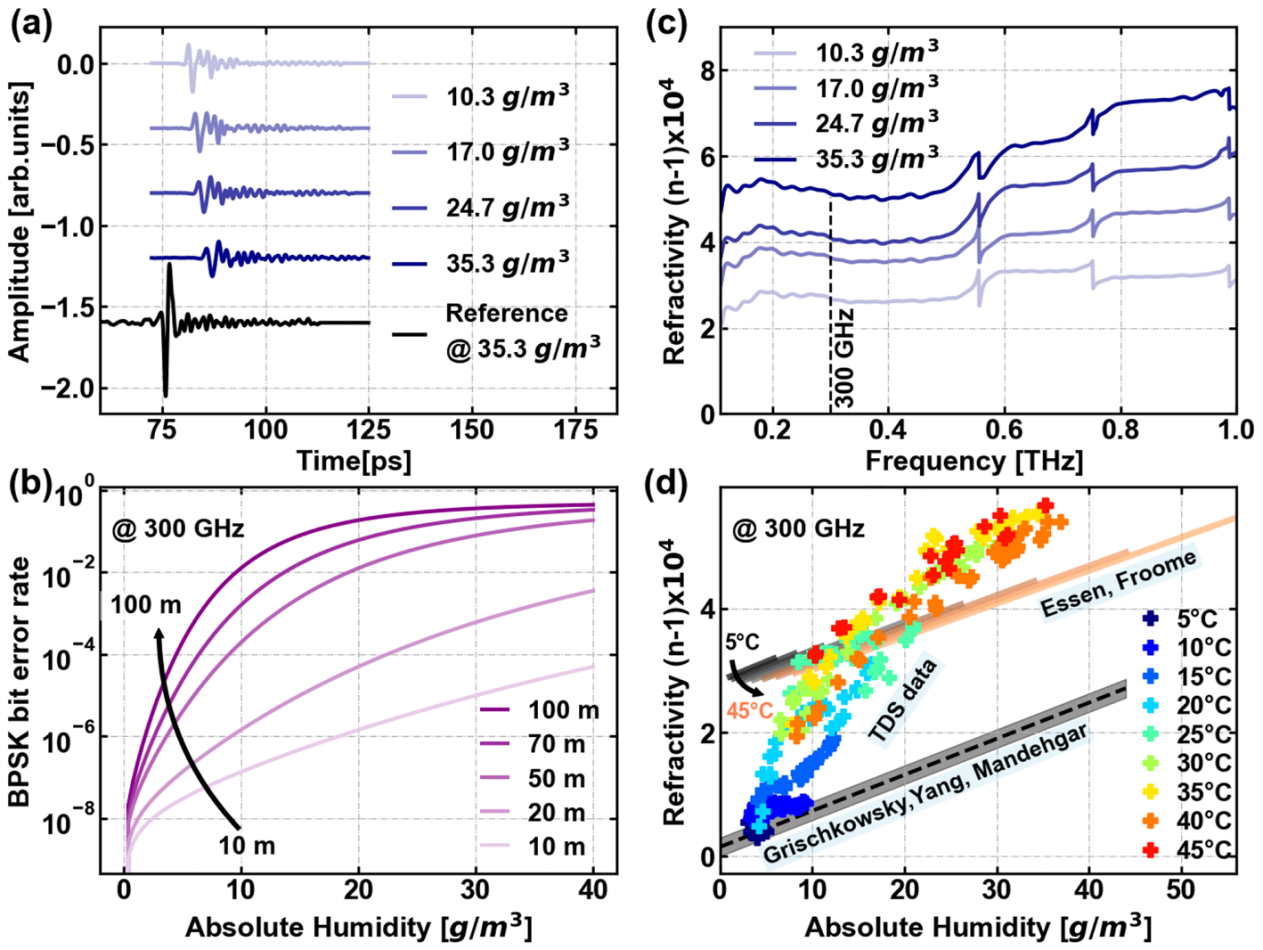
(**a**) Transmitted THz pulses at 45 °C and corresponding to 10.3 g/m$^3$, 17 g/m$^3$, 24.7 g/m$^3$, 35.3 g/m$^3$ of absolute humidity. An offset was added to each pulse for clarity. The reference pulse for 45 °C and 35.3 g/m$^3$ is shown in black as comparison. (**b**) The bit error rate (BER) at 300 GHz for BPSK modulation is evaluated as a function of absolute humidity for different distance ranges between the transmitter and receiver, ranging from 10 to 100 m. (**c**) Refractivity of water vapor at 45 °C and corresponding to 10.3 g/m$^3$, 17 g/m$^3$, 24.7 g/m$^3$, 35.3 g/m$^3$ of absolute humidity. The dashed line marks the 300 GHz frequency. (**d**) Refractivity of water vapor at a frequency of 300 GHz for the temperature range from 5 to 45 °C with step 5 °C, corresponding to a change in absolute humidity from 3 to 36.9 g/m$^3$. The refractivity values calculated using the Essen Froome model^29,37^ are represented by gray–orange palette, while the values obtained by the Grischkowsky group^44,45^ are depicted in gray.

Beyond measurements of the variation of the absorption strength near resonances, our results also can be used to estimate atmospheric effects on data transmissions which are designed to exploit frequencies far from the resonances. To illustrate this, we consider a communication link operating at 300 GHz. This frequency has been identified as one of the targets for the next generation of telecommunication systems^42^. We can then use the measured attenuation at 300 GHz, extracted from our TDS experiments, to assess the signal drop at this frequency associated with the increase in absolute humidity. This attenuation rate derived from our measurements has a value of 0.008 [dB/m]/[g/m$^3$], well below that of the values quoted earlier for the resonant peaks. We envision a link employing binary phase shift keying (BPSK) modulation with a link budget margin of 15 dB signal-to-noise (SNR) in the absence of atmospheric attenuation. We compute the bit error rate for this link as a function of absolute humidity for various different propagation distances. Further details of the calculation can be found in^43^. These calculations, shown in Fig. 10b, suggest that links of a few tens of meters of propagation range can be quite robust against changes in humidity, although when the range approaches 100 m, the sensitivity to absolute humidity increases.

### THz pulse propagation and refractivity

We also studied the effects of temperature and absolute humidity on the atmospheric refractivity. The delay of the entire THz pulse is only affected by the frequency-independent refractivity of water vapor, while the THz pulse reshaping is determined by the dispersion of the refractivity (Eq. 3). Figure 10a shows some examples of THz pulses at 45 °C and absolute humidity values of 10.3 g/m$^3$, 17 g/m$^3$, 24.7 g/m$^3$, 35.3 g/m$^3$. We also show a reference pulse at 35.3 g/m$^3$, for comparison. The pulses suffer absolute humidity dependent time delays. A stronger and extended ringing also appears, due to the dispersion and absorption. The refractivity, which we obtained from the TDS experiment, for the frequency range of 0.2 to 1 THz is presented in Fig. 10c. The refractivity shows a monotonous increase as function of the humidity across the entire spectrum. Figure 10d focuses on the dependence of refractivity with temperature and absolute humidity at 300 GHz. The temperature changes from 5 to 45 °C with 5 °C steps, which corresponds to a change of absolute humidity of 2.7 g/m$^3$ to 36.9 g/m$^3$. In this figure, the refractivity obtained from TDS measurements is compared with the refractivity of the atmosphere calculated by two previous studies (Fig. 10d). First, the refractivity calculated with the Essen and Froome equation^29,37^, is shown with the grey-orange palette. This calculation relies on the partial pressure of dry air and the partial pressure of water vapor present in the atmosphere. Second, the refractivity of the atmosphere as calculated with the same phenomenological expressions used by the Grischkowsky group^44,45^, depicted as a gray area. All curves show an increase of the refractivity with absolute humidity. The changes in refractivity imply a dependence of the group velocity dispersion on the humidity which might be relevant for the design of telecommunication links at these frequencies^20,46^.

## Conclusions

We present a characterisation of the propagation properties of the atmosphere under a wider range of temperature and humidity than any previous report to date. The attenuation at frequencies corresponding to rotational resonances of the water molecule strongly depends on the absolute humidity and less strongly on the temperature. Our measurements, using two different methods agree with the predictions of the ITU-R P.676-13 model. We also provide an estimation of the bit error rate in a prototypical communication link operation in one of the transparency bands at 300 GHz. This calculation suggests that reliable links can be achieved with a broadcast range of at least tens of meters over a wide range of atmospheric conditions. Furthermore we observed that the attenuation at the absorption peaks depends linearly to the water content with negligible dependence on the temperature. As for the refractive index of air, away from absorption lines, namely at 300 GHz, we found a sub-linear dependence with the humidity which seem to be fit in the low- and high-humidity regimes two different previous studies that were inconsistent among them.

## Data Availability

The datasets and scripts used and/or analysed during the current study are available from the corresponding author on reasonable request.
